# Hypersensitivity to PACAP-38 in post-traumatic headache: a randomized clinical trial

**DOI:** 10.1093/brain/awad367

**Published:** 2023-10-21

**Authors:** Haidar M Al-Khazali, Rune H Christensen, David W Dodick, Basit Ali Chaudhry, Faisal Mohammad Amin, Rami Burstein, Håkan Ashina

**Affiliations:** Harvard Medical School, Boston, MA 02115, USA; Department of Anesthesia, Critical Care and Pain Medicine, Beth Israel Deaconess Medical Center, Boston, MA 02115, USA; Department of Neurology, Danish Headache Center, Copenhagen University Hospital—Rigshospitalet, Copenhagen 2600, Denmark; Department of Clinical Medicine, Faculty of Health and Medical Sciences, University of Copenhagen, Copenhagen 2100, Denmark; Harvard Medical School, Boston, MA 02115, USA; Department of Anesthesia, Critical Care and Pain Medicine, Beth Israel Deaconess Medical Center, Boston, MA 02115, USA; Department of Neurology, Danish Headache Center, Copenhagen University Hospital—Rigshospitalet, Copenhagen 2600, Denmark; Department of Clinical Medicine, Faculty of Health and Medical Sciences, University of Copenhagen, Copenhagen 2100, Denmark; Department of Clinical Medicine, Faculty of Health and Medical Sciences, University of Copenhagen, Copenhagen 2100, Denmark; Department of Neurology, Mayo Clinic, Scottsdale, AZ 85259, USA; Department of Neurology, Danish Headache Center, Copenhagen University Hospital—Rigshospitalet, Copenhagen 2600, Denmark; Department of Clinical Medicine, Faculty of Health and Medical Sciences, University of Copenhagen, Copenhagen 2100, Denmark; Department of Neurology, Danish Headache Center, Copenhagen University Hospital—Rigshospitalet, Copenhagen 2600, Denmark; Department of Clinical Medicine, Faculty of Health and Medical Sciences, University of Copenhagen, Copenhagen 2100, Denmark; Department of Brain and Spinal Cord Injury, Copenhagen University Hospital—Rigshospitalet, Copenhagen 2600, Denmark; Harvard Medical School, Boston, MA 02115, USA; Department of Anesthesia, Critical Care and Pain Medicine, Beth Israel Deaconess Medical Center, Boston, MA 02115, USA; Harvard Medical School, Boston, MA 02115, USA; Department of Anesthesia, Critical Care and Pain Medicine, Beth Israel Deaconess Medical Center, Boston, MA 02115, USA; Department of Neurology, Danish Headache Center, Copenhagen University Hospital—Rigshospitalet, Copenhagen 2600, Denmark; Department of Clinical Medicine, Faculty of Health and Medical Sciences, University of Copenhagen, Copenhagen 2100, Denmark; Department of Brain and Spinal Cord Injury, Copenhagen University Hospital—Rigshospitalet, Copenhagen 2600, Denmark

**Keywords:** headache disorders, migraine, trigeminovascular system, pathophysiology, concussion

## Abstract

Pituitary adenylate cyclase-activating polypeptide-38 (PACAP-38), known for its role in migraine pathogenesis, has been identified as a novel drug target. Given the clinical parallels between post-traumatic headache (PTH) and migraine, we explored the possible role of PACAP-38 in the pathogenesis of PTH. To this end, we conducted a randomized, double-blind, placebo-controlled, two-way crossover trial involving adult participants diagnosed with persistent PTH resulting from mild traumatic brain injury.

Participants were randomly assigned to receive a 20-min continuous intravenous infusion of either PACAP-38 (10 pmol/kg/min) or placebo (isotonic saline) on two separate experimental days, with a 1-week washout period in between. The primary outcome was the difference in incidence of migraine-like headache between PACAP-38 and placebo during a 12-h observational period post-infusion. The secondary outcome was the difference in the area under the curve (AUC) for baseline-corrected median headache intensity scores during the same 12-h observational period.

Of 49 individuals assessed for eligibility, 21 were enrolled and completed the trial. The participants had a mean age of 35.2 years, and 16 (76%) were female. Most [19 of 21 (90%)] had a migraine-like phenotype. During the 12-h observational period, 20 of 21 (95%) participants developed migraine-like headache after intravenous infusion of PACAP-38, compared with two (10%) participants after placebo (*P* < 0.001). Furthermore, the baseline-corrected AUC values for median headache intensity scores during the 12-h observational period was higher after PACAP-38 than placebo (*P* < 0.001).

These compelling results demonstrate that PACAP-38 is potent inducer of migraine-like headache in people with persistent PTH. Thus, targeting PACAP-38 signalling might be a promising avenue for the treatment of PTH.

## Teaser

Al-Khazali *et al.* show in a randomized, double-blind, placebo-controlled trial that intravenous infusion of PACAP-38 is a potent inducer of migraine-like headache in people with persistent post-traumatic headache (PTH) resulting from mild traumatic brain injury. Targeting PACAP-38 signalling could be a promising therapeutic approach for PTH.

## Introduction

Post-traumatic headache (PTH) is a disabling neurological disorder that affects millions of people worldwide and is often attributed to mild traumatic brain injury (TBI).^1,2^ The clinical presentation of PTH is characterized by recurrent episodes of headache that tend to resemble migraine.^3^ Furthermore, PTH is often accompanied by comorbid symptoms suggestive of sleep disturbances, anxiety, depression and cognitive impairment.^4^ Despite a high prevalence,^5^ there are currently no approved medications for PTH, leaving a considerable unmet treatment need.^6^

The trauma that results in PTH can stem from both direct and indirect impact to the head, possibly causing sensitization of trigeminal sensory afferents that innervate pain-sensitive intracranial structures, such as the meninges and its blood vessels.^7^ The initial mechanisms posited to activate meningeal nociceptors involve the release of various signalling molecules from sensory afferents and parasympathetic efferents.^7^ These include calcitonin gene-related peptide (CGRP) and pituitary adenylate cyclase-activating polypeptide-38 (PACAP-38).^8^ Evidence from human experimental studies have shown that intravenous infusion of CGRP can induce migraine attacks in people with migraine and migraine-like headache in those with persistent PTH.^9-11^

An outstanding scientific question is whether PACAP-38, as seen with CGRP, can induce migraine-like headache in people with persistent PTH. In this context, it merits emphasis that intravenous infusion of PACAP-38 elicits migraine attacks in people with migraine, while healthy individuals experience mild or no headache.^12^ Furthermore, recent phase II results demonstrated the effectiveness of a monoclonal antibody (mAb) against PACAP signalling for migraine prevention.^13^ If it is confirmed that PACAP-38 can indeed induce migraine-like headache in people with PTH, this will bolster the concept of targeting PACAP signalling. It potentially opens a novel avenue for therapeutic intervention, specifically for the preventive treatment of PTH.

Here, we report the results of a randomized, double-blind, placebo-controlled, two-way crossover trial that evaluated the incidence of migraine-like headache after intravenous infusion of PACAP-38 in people with persistent PTH who had no history of a primary headache disorder, including migraine.

## Materials and methods

The study protocol was approved by the Regional Health Research Ethics Committee of the Capital Region of Denmark and all participants provided written informed consent before the commencement of any study-related assessments or procedures. The study was registered with ClinicalTrials.gov (identifier: NCT05378061) and conducted in accordance with the principles of the Declaration of Helsinki. Participants were enrolled from the outpatient clinic of a tertiary care unit during the period from June 2022 to September 2022. The full trial protocol is available in the Supplementary material, ‘Appendix’.

All authors vouch for the accuracy and completeness of the data, and both the first and senior author had unrestricted access to the data and drafted the initial version of the manuscript, which was revised and edited by all authors.

### Participants

Eligible participants were 18 to 65 years of age and had a diagnosis of persistent PTH attributed to mild TBI in accordance with the third edition of the International Classification of Headache Disorders (ICHD-3).^14^ Participants were also required to report at least four monthly headache days, as an average across the 3 months prior to enrolment. The main exclusion criterion was any history of a primary or secondary headache disorder prior to the mild TBI, except for infrequent episodic tension-type headache.^14^ Additional exclusion criteria were any history of more than one mild TBI, moderate-to-severe TBI, whiplash injury, or craniotomy. Participants were also excluded if they had initiated, discontinued or changed the dosing of preventive headache medication within 2 months prior to enrolment. A review of electronic medical records was performed to further ensure eligibility for study inclusion. The full list of eligibility criteria is provided in Supplementary Tables 1 and 2.

### Design

The present trial had a randomized, double-blind, placebo-controlled, two-way crossover design and was conducted at a single site. Independent pharmacy staff were responsible for drug preparation, randomization and allocation concealment. Block randomization was used, with each block comprising four participants: two received PACAP-38 infusion and two received placebo. The sequence within blocks was determined using a random number generator to ensure blinding. Of the 21 participants, five blocks contained four participants each, with a single participant in the final block. Participants were randomly assigned to receive continuous intravenous infusion of 20 ml PACAP-38 (10 pmol/kg/min) or 20 ml placebo (isotonic saline) in the antecubital fossa for 20 min on two experimental days. A time and volume-controlled infusion pump was used for this purpose and the two experimental days were separated by a washout period of at least 7 days. PACAP-38 was administered at a dose identical to that used in previous human experimental studies.^15,16^

Participants were rescheduled for another experimental day if they reported intake of acute medications within 48 h of the planned infusion start, had a baseline headache intensity of more than 3 on the 11-point numeric rating scale (0 being no headache, 10 being the worst imaginable headache), or complained of migraine-like headache at the time of infusion start.

### Procedure

Upon arrival on the first experimental day, site investigators collected data on demographics, medical history and full clinical course. A physical and neurological examination was subsequently performed, and the participants were informed that PACAP-38 might induce headache, but no details were provided about onset, duration or features. The same procedures were then repeated on both experimental days. Participants were placed in a supine position and the antecubital fossa was cannulated to obtain peripheral intravenous access. At baseline (i.e. the time of infusion start), the site investigator recorded data on clinical features, intake of rescue medication, adverse events and vital signs (i.e. blood pressure and heart rate) using a 12-h headache diary. This procedure was repeated every 10 min until 60 min after the start of infusion. Participants were then discharged and instructed to complete the headache diary hourly until 12 h after the infusion started.

### Statistical analysis

Descriptive statistics are presented using means ± standard deviations (SD) for normally distributed data, while skewed data are described using medians and interquartile ranges (IQR). As an exception, monthly headache days are presented using means ± SD, aligning with the conventional presentation in literature on headache disorders. The normal distribution was evaluated by visual inspection and using the Shapiro-Wilk test. Table 1 also provides the corresponding median and IQR values. Categorical data were presented with absolute numbers and percentages. Analyses for the primary and secondary outcomes involved all randomly assigned participants in adherence with the intention-to-treat principle.

**Table 1:** Demographic and clinical characteristics of the study population

Characteristics	Persistent PTH, *n* = 21
Age, mean ± SD, years	35.2 ± 11.0
Sex, *n* (%)	
Male	5 (23.8)
Female	15 (76.2)
Body mass index, mean ± SD, kg/m^2^	24.3 ± 4.3
Family history of migraine without aura, *n* (%)	1 (4.8)
Family history of migraine with aura, *n* (%)	2 (9.5)
Time since TBI, median (IQR), years	8.1 (3.2–13.0)
Migraine-like phenotype, *n* (%)	19 (90.5)
Tension-type headache-like phenotype, *n* (%)	2 (9.5)
Monthly headache days, mean ± SD	26.4 (7.6)
Current use of acute headache medication, *n* (%)	17 (81.0)
Current use of preventive headache medication, *n* (%)	10 (47.6)

IQR = interquartile range; *n* = number; PTH = post-traumatic headache; SD = standard deviation; TBI = traumatic brain injury.

The primary outcome was the difference in incidence of migraine-like headache between PACAP-38 and placebo during the 12-h observational period after infusion start. The analysis was conducted using McNemar’s test, which can be used to evaluate differences between paired groups reporting binary outcomes. Sample size calculations were based on the primary outcome and a one-sided McNemar’s test was used for this purpose. A sample size of 21 participants was needed to achieve 80% power at a significance level of 5%, assuming that 50% of participants report migraine-like headache exclusively after PACAP-38 and 10% exclusively after placebo. The sample size calculation was conducted in R (version 4.2.3). The criteria for experimentally-induced migraine-like headache is shown in Box 1. The secondary outcome was the difference in area under the curve (AUC) for median headache intensity scores between PACAP-38 and placebo during the 12-h observational period after infusion start. The AUC values were baseline-corrected and used as a summary measure in adherence with the trapezium rule. The baseline correction was applied to reduce the influence of baseline headache intensity scores between the two experimental days. The results for the secondary outcome were compared using the Wilcoxon signed-rank test. In addition, the percentage change in mean arterial blood pressure and heart was calculated and summarized using AUC values. The results for PACAP-38 and placebo were then compared using a paired *t*-test. Moreover, testing for carryover effects was carried out by using a binomial regression model in R to assess the potential influence of the intervention (PACAP-38 or placebo) received on the first experimental day on the outcomes observed on the second experimental day. All statistical analyses were performed using Microsoft Excel (v2102) and R (v4.1.0).

Box 1 **Criteria for experimentally-induced migraine-like headache**

The following criteria are used for experimentally induced migraine-like headache:

Migraine-like headache must fulfil at least two of the following four characteristics:

- Unilateral location
- Pulsating quality
- Moderate or severe pain intensity
- Aggravation by or causing avoidance of routine physical activity (e.g. walking or climbing stairs)

During headache, at least one of the following must be fulfilled:

- Nausea and/or vomiting
- Photophobia and phonophobia, or
- Headache mimicking the usual headache exacerbation with migraine-like features

## Results

### Participants

**Figure 1:**
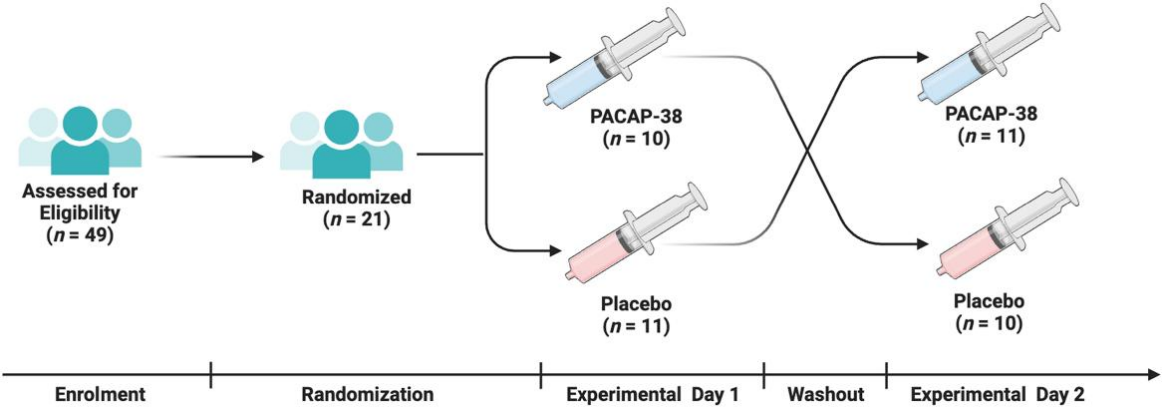
**Study flow diagram**. *n* = number; PACAP-38 = pituitary adenylate cyclase-activating polypeptide-38.

A total of 49 people were screened for eligibility from June 2022 to September 2022. Among them, 21 participants were enrolled and completed the trial (Fig. 1). The enrolled participants had a mean age of 35.2 ± 11.0 years, and most were female [16 of 21 (76%)]. The median number of years since the mild TBI was 8.1 (IQR, 3.2 to 13.0) and the mean number of monthly headache days was 26.4 ± 7.6. Among the participants, 19 (90%) of 21 reported a migraine-like phenotype and 3 of 21 (14%) had a family history of migraine with or without aura. About half of the participants [10 of 21 (48%)] reported current use of preventive headache medications. The demographic and clinical characteristics of the participants are summarized in Table 1.

### Migraine-Like headache and headache intensity scores

Intravenous infusion of PACAP-38 induced migraine-like headache in 20 (95%) of 21 participants with persistent PTH, compared with two (10%) participants after placebo (*P* < 0.001; Supplementary Table 3). No carryover effects were observed between PACAP-38 and placebo (*P* = 0.54) (Supplementary Table 4). Furthermore, 18 of 21 participants developed migraine-like headache exclusively after intravenous infusion of PACAP-38, whilst none did so after placebo. During the 60-min in-hospital period, 12 of 21 participants reported migraine-like headache after PACAP-38, compared with one participant after placebo.

**Figure 2:**
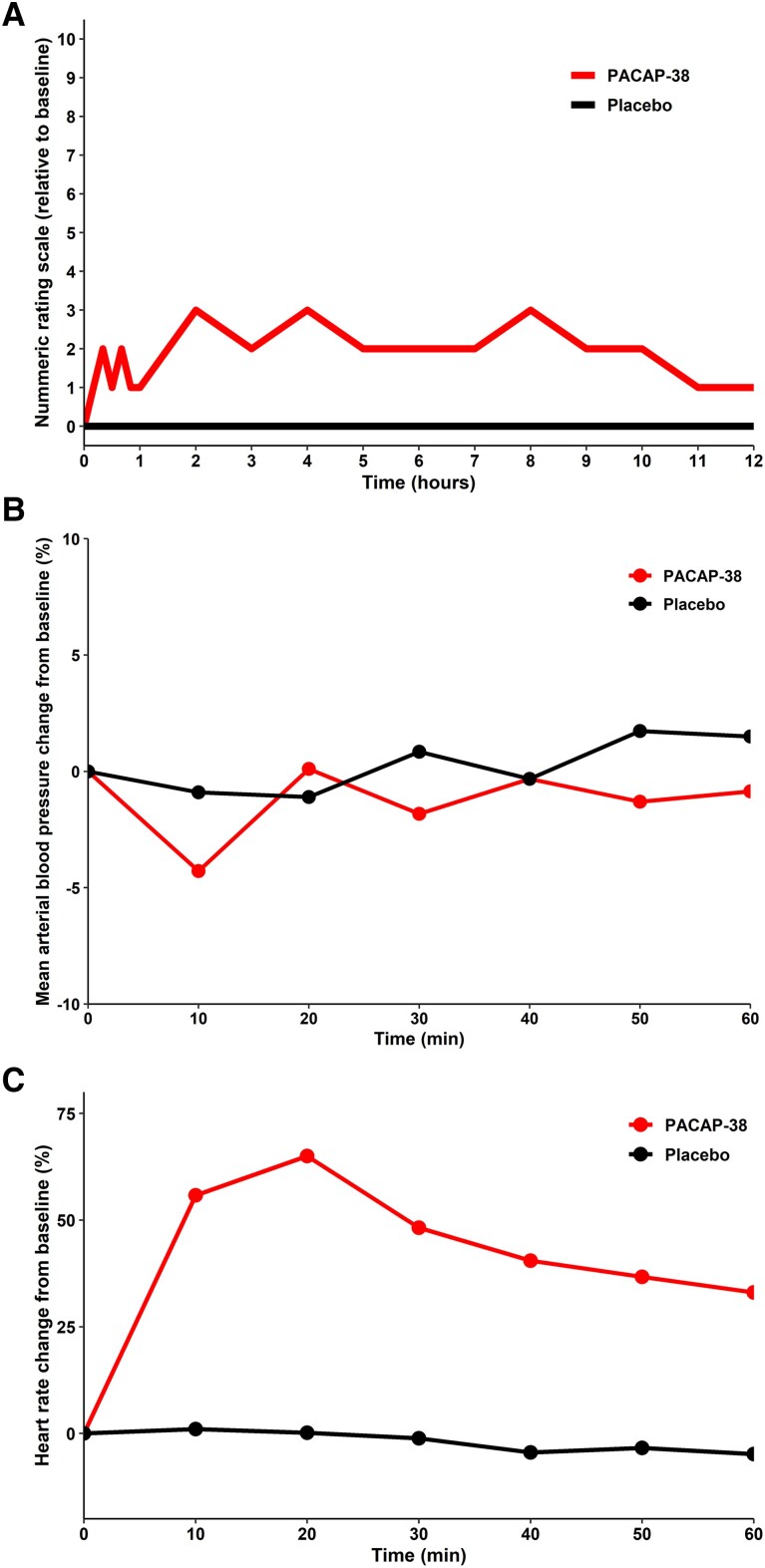
**Headache scores and haemodynamic variables**. (**A**) Baseline-corrected median headache intensity scores after PACAP-38 and placebo infusion during the 12-h observational period. The baseline headache intensity scores were subtracted before calculating area under the curve (AUC) to reduce influence of within-participant variations in headache at baseline between the two experimental days. The red line denotes median headache intensity scores after PACAP-38, whilst the black line denotes median headache intensity scores after placebo. (**B**) Baseline-corrected mean arterial blood pressure values after PACAP-38 and placebo infusion during the 60-min in-hospital period. The red line denotes mean arterial blood pressure after PACAP-38, whilst the black line denotes mean arterial blood pressure after placebo. (**C**) Baseline-corrected mean heart rate values after PACAP-38 and placebo infusion during the 60-min in-hospital period. The red line denotes mean heart rates after PACAP-38, whilst the black line denotes mean heart rates after placebo. PACAP-38 = pituitary adenylate cyclase-activating polypeptide-38.

The baseline-corrected AUC for median headache intensity scores were higher after PACAP-38 compared with placebo (*P* < 0.001; Fig. 2A). There were no carryover effects observed between PACAP-38 and placebo (*P* = 0.717) (Supplementary Table 4). The median peak headache intensity was 6 (IQR, 5 to 7) following PACAP-38 and 3 (IQR, 3 to 4) after placebo. Furthermore, the median time to peak headache intensity was 180 min (IQR, 40 to 300 min) following PACAP-38 and 90 min (IQR, 0 to 420 min) after placebo. The peak headache was primarily of bilateral location [*n* = 17 (81%)], pressing quality [*n* = 10 (48%)], and moderate-to-severe pain intensity [*n* = 19 (90%)]. In addition, most participants experienced pain aggravation by routine physical activity [*n* = 14 (67%)] and accompanying photophobia [*n* = 17 (81%)], phonophobia [*n* = 12 (57%)] and nausea or vomiting [*n* = 14 (67%)] (Supplementary Fig. 1).

### Adverse events and use of rescue medication

The reported adverse events were palpitations (*n* = 20 after PACAP-38 versus *n* = 1 after placebo, *P* < 0.001), flushing (*n* = 21 after PACAP-38 versus *n* = 0 after placebo, *P* < 0.001), and warm sensations (*n* = 21 after PACAP-38 versus *n* = 2 after placebo, *P* < 0.001). After receiving PACAP-38 infusion, 18 of 21 participants used rescue medication to treat their headache, whilst three did so after placebo.

### Haemodynamic variables

During the 60-min in-hospital period, the percentage change in baseline-corrected AUC for mean arterial blood pressure did not differ between PACAP-38 and placebo (*P* = 0.2; Fig. 2B). The corresponding percentage change in baseline-corrected AUC for mean heart rate was higher after PACAP-38, compared with placebo (*P* < 0.001; Fig. 2C and Supplementary Table 5).

## Discussion

This study presents a novel finding that PACAP-38 infusion can induce migraine-like headache in people with persistent PTH, which closely resemble their typical episodes of migraine-like headache. The implications of this study are 2-fold. First, people with persistent PTH are hypersensitive to PACAP-38, as healthy individuals free of headaches report only mild or no headache following PACAP-38 infusion.^12^ Second, our findings support the hypothesis that PACAP signalling plays a significant role in the genesis of migraine-like headache in PTH.

Ample human experimental data support the involvement of PACAP-38 in cephalic pain.^12,15,16^ In a randomized, double-blind, placebo-controlled, two-way crossover trial,^12^ intravenous infusion of PACAP-38 induced migraine attacks in seven (58%) of 12 participants with migraine, compared with none after placebo. The same study revealed that PACAP-38 induced mild headache in healthy individuals.^12^ Furthermore, recent phase II trial data demonstrated the effectiveness of a mAb against PACAP signalling for migraine prevention.^13^ Considering the common occurrence of migraine-like headache in people with PTH and our finding of hypersensitivity to PACAP-38 in this patient population, blocking PACAP-38 effects might serve as a mechanism-based treatment option for PTH.

### PACAP-38 in provoked migraine-like headache

**Figure 3:**
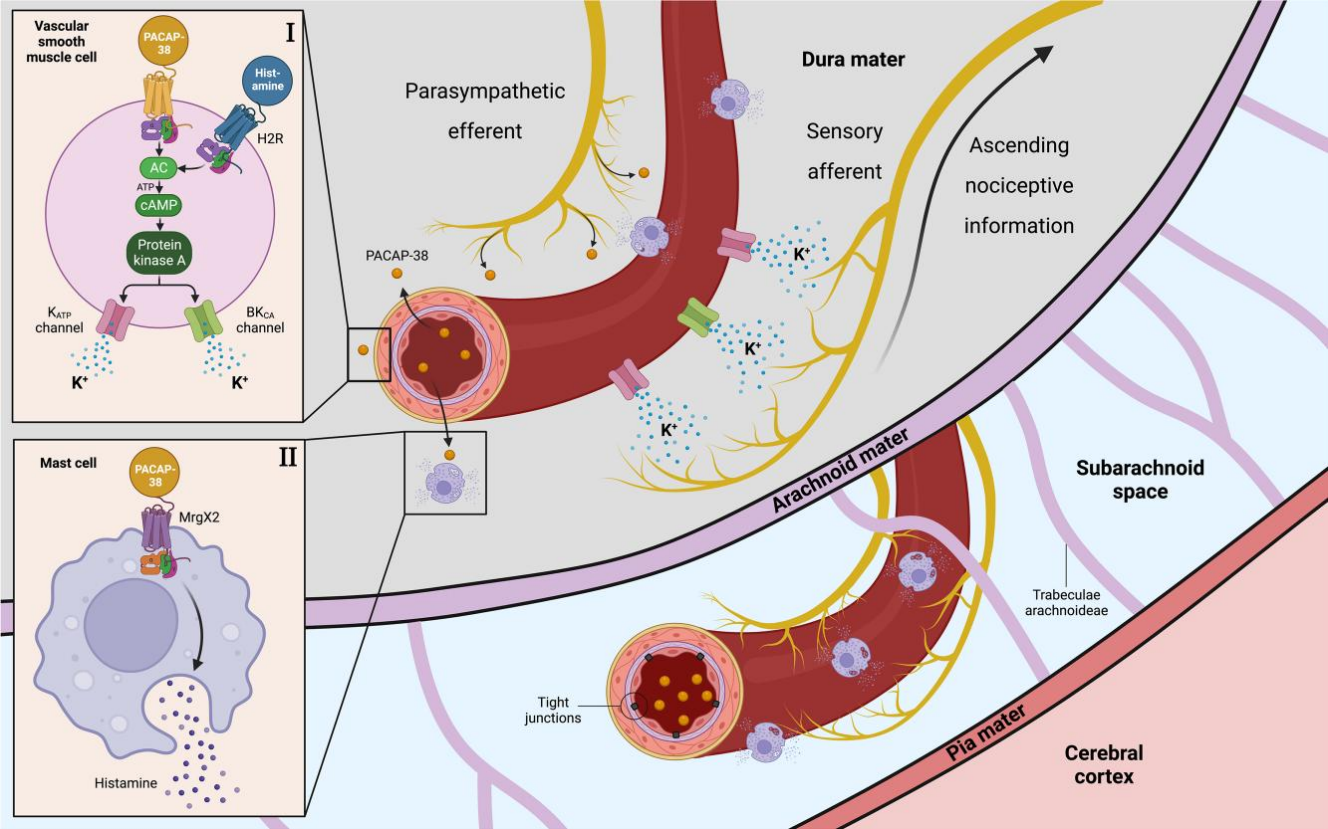
**Possible mechanisms and sites of action of PACAP-38-induced migraine-like headache in people with persistent post-traumatic headache.** The figure illustrates two possible sites of action of PACAP-38 induced migraine-like headache. (I) Indirect activation of meningeal nociceptors occurs through the dilation of intracranial arteries, accompanied by the potassium efflux from vascular smooth muscle cells. PACAP-38 binds to its G protein-coupled receptor in the vascular smooth muscle cell, initiating the cAMP-dependent signalling pathway. This pathway leads to the opening of K_ATP_ channels and BK_Ca_ channels, resulting in potassium efflux and subsequent vasodilation. These processes are hypothesized to activate and sensitize perivascular meningeal nociceptors through chemical and mechanical stimulation. (II) Indirect activation of meningeal nociceptors is mediated by mast cell degranulation and the release of pro-nociceptive chemical agents. PACAP-38 binds to its MrgX2 receptor in mast cells, causing degranulation of histamine and tryptase. Both histamine and tryptase bind to their respective G protein-coupled receptors on vascular smooth muscle cells, leading to vasodilation and activation of meningeal nociceptors, likely through a similar mechanism as described in scenario (I). AC = adenylate cyclase; ATP = adenosine triphosphate; BK_Ca_ channels = large conductance calcium-activated potassium channel; cAMP = cyclic adenosine monophosphate; H2R = histamine receptor H2; KATP channels = ATP-sensitive potassium channels; PACAP-38 = pituitary adenylate cyclase-activating polypeptide-38.

The exact mechanisms and sites of action that underlie the induction of cephalic pain, specifically migraine-like headache, by intravenous infusion of PACAP-38 are not yet fully understood. We propose the following two potential mechanisms (Fig. 3): (i) the indirect activation of meningeal nociceptors by the dilation of intracranial arteries and the accompanying efflux of potassium from vascular smooth muscle cells (VSMCs); (ii) the indirect activation of meningeal nociceptors via mast cell degranulation and the accompanying release of pro-nociceptive chemical agents. Although less likely, other mechanisms include; (iii) direct activation of meningeal nociceptors by PACAP-38 binding to its receptors on primary afferents; and (iv) direct activation of neurons in the trigeminocervical complex (TCC) through PACAP-38 binding to its receptors on these neurons.

The intravenous infusion of PACAP-38 is known to dilate meningeal arteries, including the middle meningeal artery (MMA), in both people with migraine and healthy individuals.^15,17^ This dilation is initiated by binding of PACAP-38 to its G protein-coupled receptors on the VSMCs within the walls of the meningeal arteries.^18,19^ This binding upregulates intracellular levels of cyclic adenosine monophosphate (cAMP), causing activation of protein kinase A.^20^ The latter then phosphorylates and increases the probability for opening of ATP-sensitive potassium channels (K_ATP_ channels) and large conductance calcium-activated potassium (BK_Ca_) channels,^21,22^ which leads to the efflux of positively charged potassium ions. The resulting membrane hyperpolarization and decreased calcium entry ultimately yield vasodilation.^21,22^

Regarding the pathogenesis of cephalic pain, randomized placebo-controlled trials have shown that the upregulation of cAMP and the opening of K_ATP_ and BK_Ca_ channels are potent inducers of migraine attacks in people with migraine and mild headache in healthy individuals.^23-26^ The exact mechanism by which the arterial dilation and efflux of potassium translate into nociception is not fully understood. A 2020 hypothesis suggests that these events might provide the necessary mechanical and chemical stimuli to activate and sensitize meningeal nociceptors in the perivascular space.^27^ Nociceptive information is then relayed to the somatosensory cortex and other cortical/subcortical areas responsible for pain perception via second-order TCC neurons and third-order thalamic neurons.^27^ Further studies are needed to confirm this sequence of events. Such efforts might include *in vivo* electrophysiological recordings of neuronal firing rates at the level of meningeal nociceptors, as well as second-order TCC neurons and third-order thalamic neurons, following the administration of PACAP-38 in rodents.

The intravenous infusion of PACAP-38 might also induce cephalic pain in people with PTH through mast cell degranulation, activating meningeal nociceptors.^28,29^ Mast cells, abundant in the meninges, play a key role in inflammatory responses.^29,30^ PACAP-38 is thought to trigger mast cell degranulation in humans, likely via its MRGX2 receptor.^31^ This degranulation releases histamine, a pro-nociceptive agent that induces migraine attacks and dilates meningeal arteries.^32,33^ The precise role of histamine in PACAP-38-induced dilation of meningeal arteries is uncertain. However, studies have shown that pretreatment with the antihistamine clemastine reduces migraine attacks after PACAP-38 infusion.^34^ Animal research also demonstrates reduced MMA dilation in mast cell-depleted rodents and those given antihistamines.^35^ In addition to histamine, PACAP-induced mast cell degranulation might release tryptase, a pro-inflammatory agent that interacts with protease-activated receptor 2 (PAR2) on the VMSCs.^36^ PAR2 activation initiates signalling pathways linked to vasodilation and sensitization of meningeal nociceptors.^36-38^ Recent studies highlight PAR2 as a promising therapeutic target for headache disorders.^39^ Taken together, the current evidence suggests that PACAP-38 can directly bind to its receptors on VSMCs, leading to arterial dilation, and also promote histamine as well tryptase release from mast cells, which then cause arterial dilation after binding to their receptors on the VSMCs. In both instances, the vasodilation and accompanying potassium efflux might provide the necessary mechanical and chemical stimuli to sensitize and activate the meningeal nociceptors.^26^

There are other, less likely, mechanisms and sites of action through which PACAP-38 might induce migraine-like headache. One option is that PACAP-38 directly activates first-order neurons located in the trigeminal ganglion and upper cervical ganglia. Yet, this appears less probable, drawing upon evidence from *in vivo* rodent experiments that recorded the firing rates of second-order TCC neurons.^40^ These experiments revealed that PACAP-38, when administered intravenously, incited nociceptive responses. However, antagonists of VPAC_1/2_ and PAC_1_ receptors did not produce inhibitory effects on nociceptive responses. The inference drawn was that PACAP-38 might cross the blood–brain barrier, exerting direct effects on structures within the CNS.^40^ In support, intracerebroventricular administration of both VPAC_1_ and PAC_1_ receptor antagonists diminished nociceptive responses in second-order TCC neurons of rodents.^40^ These results paved the way for postulating that PACAP-38 might exert a direct effect on second-order TCC neurons. Yet, it seems improbable that PACAP-38 can cross the blood–brain barrier, considering its molecular size. Rodent data involving radiolabelled PACAP identified a mere brain uptake of <0.07% for both its isoforms (i.e. PACAP-38 and PACAP-27) in rodents.^41^ Recent findings also cast further doubt on the therapeutic promise of PAC_1_ receptor antagonism, given a monoclonal antibody against the PAC1 receptor failed against placebo for migraine prevention in a phase II trial.^42^ Nonetheless, concerns with dosing, potency, and the possibility of alternative receptor splice variants, cannot be excluded as reasons for failure. It can therefore not be entirely ruled out that PACAP-38 induced migraine-like headache might be mediated via direct binding to its receptors on first-order or possibly even second-order neurons.

#### Temporal patterns

An intriguing observation warranting discussion is the time elapsed from the start of PACAP-38 infusion to the onset of migraine-like headache. Despite PACAP-38’s direct administration into the circulation, participants reached peak headache intensity at varying intervals, with a median time of 180 min post-infusion. The disparate observations might underscore intra- and interindividual variation in sensitivities to PACAP-38. It is conceivable that specific sensory thresholds exist at an individual level, activating nociceptive pathways leading to PACAP-38 induced migraine-like headache. In addition to this, the intricate relationship between PACAP-38’s downstream effects and the modulation of nociception by other inflammatory mediators, such as CGRP, might be contributing factors. Further investigation is needed to discern the mechanisms underpinnings the observed temporal patterns from PACAP-38 infusion to the onset of migraine-like headache.

#### PACAP-38: responses in post-traumatic headache versus migraine

The intravenous infusion of PACAP-38 is recognized to induce migraine attacks in people diagnosed with migraine without aura.^12,15^ This background serves as an interesting context to our findings. For instance, one randomized clinical trial reported that PACAP-38 infusion induced migraine attacks in 7 of 12 (58%) participants with migraine, compared with none after placebo.^12^ Their reported median peak headache intensity stood at 2.5 (range, 0–10). In contrast, our results showed that 20 of 21 (95%) participants with persistent PTH experienced migraine-like headache after PACAP-38 infusion, recording a median peak headache intensity of 6 (range, 3–9). However, it is critical to recognize the distinct context. Our study included participants with persistent PTH, averaging 26.4 monthly headache days and permitted to report a mild headache at the time of infusion start. The migraine group, on the other hand, experienced between one and four monthly attacks and had no headache at the start of infusion. Hence, it is advisable to steer clear of direct comparisons. Rather, the emphasis should be on PACAP-38’s superior ability over placebo in eliciting migraine-like headache.

### Targeting PACAP signalling in post-traumatic headache

The absence of evidence-based treatments for PTH represents a glaring therapeutic gap, underscoring the need for identifying novel drug targets. In this context, our results provide compelling evidence supporting the potential of targeting PACAP signalling as a promising avenue for drug discovery in PTH. One important aspect to emphasize is the growing body of evidence indicating that PACAP-38 acts through distinct sites and mechanisms compared with CGRP,^8,43^ including its involvement in mast cell degranulation.^44^ This observation suggests that drugs targeting PACAP signalling might offer a differentiated approach compared with existing drugs targeting CGRP signalling, which have shown limited effectiveness in treating PTH.^45,46^ Furthermore, animal studies have indicated that CGRP has a more prominent role in the acute stage of PTH,^47^ whilst it is possible that PACAP-38 has broader involvement in both the acute and persistent stages of the disorder. Thus, elucidating the involvement of PACAP in the neurobiological underpinnings of PTH holds promise for the development of novel drugs that can address the unmet needs of those affected.

### Limitations

This study has several limitations. First, the in-hospital observational period was limited to 1-h post-infusion for feasibility purposes, and participants were then instructed to record outcome data every hour from 1 h to 12 h post-infusion. Therefore, the incidence of migraine-like headache might have been influenced by environmental factors such as stress, certain foods, or specific activities. Nonetheless, most participants reported the onset of migraine-like headache during the in-hospital phase. Second, the inclusion of participants with a mild headache at the time of infusion start might be perceived as a confounder. One can speculate that such participants might be more susceptible to develop a migraine-like headache after PACAP-38 infusion. Nonetheless, it is important to put this into the clinical context: people with persistent PTH often report near-daily or even continuous and unremitting headache. Hence, imposing a criterion of being entirely headache-free at the time of infusion start might unduly narrow the participant profile, thereby failing to capture the diversity seen in clinical practice. Moreover, all three participants who were headache-free at the start of the PACAP-38 infusion developed a migraine-like headache during the ensuing 12-h observational period. This underscores the robustness of our findings, albeit future studies might still benefit from considering the potential influence of headache intensity at the time of infusion start on the observed effects. Third, the present study did not investigate the sites and mechanisms of action by which PACAP-38 induces migraine-like headache. To determine these, animal investigations with *in vivo* electrophysiological recordings in rodents can be employed. Some pathophysiological insights might also be obtained from the combined use of human experimental studies with intravenous infusion of PACAP-38 and advanced functional or metabolic MRI. Fourth, our findings cannot be generalized to people with acute PTH as the present study only enrolled people with persistent PTH. Further studies are needed to determine whether people with acute PTH also exhibit hypersensitivity to PACAP-38 signalling. Last, while our findings suggest a role for PACAP-38 signalling in the pathogenesis of PTH, one must differentiate between exogenously administered PACAP-38 and its endogenous concentrations. The exact role of endogenous PACAP-38 in the pathogenesis of spontaneous episodes with migraine-like headache is yet to be elucidated.

## Conclusions

Among people with persistent PTH, intravenous infusion of PACAP-38 induced migraine-like headache in 20 of 21 (95%) participants, possibly indicating a high sensitivity to PACAP signalling in this patient population. This novel discovery suggests that targeting PACAP signalling might be a promising therapeutic approach for treating PTH.

## Supplementary Material

awad367_Supplementary_Data

## Data Availability

The data that support the findings of this study are available from the corresponding author, upon reasonable request.

## References

[1] Ashina H , Eigenbrodt AK, Seifert T, et al Post-traumatic headache attributed to traumatic brain injury: Classification, clinical characteristics, and treatment. Lancet Neurol. 2021;20:460–469. 34022171 10.1016/S1474-4422(21)00094-6

[2] Ashina H , Porreca F, Anderson T, et al Post-traumatic headache: Epidemiology and pathophysiological insights. Nat Rev Neurol. 2019;15:607–617. 31527806 10.1038/s41582-019-0243-8

[3] Ashina H , Iljazi A, Al-Khazali HM, et al Persistent post-traumatic headache attributed to mild traumatic brain injury: Deep phenotyping and treatment patterns. Cephalalgia. 2020;40:554–564. 32102546 10.1177/0333102420909865

[4] Ashina H , Al-Khazali HM, Iljazi A, et al Psychiatric and cognitive comorbidities of persistent post-traumatic headache attributed to mild traumatic brain injury. J Headache Pain. 2021;22:83. 34311696 10.1186/s10194-021-01287-7 PMC8314480

[5] Nampiaparampil DE . Prevalence of chronic pain after traumatic brain injury: A systematic review. JAMA. 2008;300:711–719. 18698069 10.1001/jama.300.6.711

[6] Larsen EL , Ashina H, Iljazi A, et al Acute and preventive pharmacological treatment of post-traumatic headache: A systematic review. J Headache Pain. 2019;20:98. 31638888 10.1186/s10194-019-1051-7 PMC6802300

[7] Ashina H , Moskowitz MA. Shared biological foundations of post-traumatic headache and migraine. Headache. 2021;61:558–559. 33634463 10.1111/head.14084

[8] Kuburas A , Russo AF. Shared and independent roles of CGRP and PACAP in migraine pathophysiology. J Headache Pain. 2023;24:34. 37009867 10.1186/s10194-023-01569-2 PMC10069045

[9] Iljazi A , Ashina H, Zhuang ZA, et al Hypersensitivity to calcitonin gene-related peptide in chronic migraine. Cephalalgia. 2021;41:701–710. 33322922 10.1177/0333102420981666

[10] Ashina H , Iljazi A, Al-Khazali HM, et al Hypersensitivity to calcitonin gene–related peptide in post-traumatic headache. Ann Neurol. 2020;88:1220–1228. 32959458 10.1002/ana.25915

[11] Ashina H , Iljazi A, Al-Khazali HM, et al CGRP-induced migraine-like headache in persistent post-traumatic headache attributed to mild traumatic brain injury. J Headache Pain. 2022;23:135. 36253732 10.1186/s10194-022-01499-5 PMC9578273

[12] Schytz HW , Birk S, Wienecke T, Kruuse C, Olesen J, Ashina M. PACAP38 Induces migraine-like attacks in patients with migraine without aura. Brain. 2009;132:16–25. 19052139 10.1093/brain/awn307

[13] Lundbeck. Lundbeck announces positive phase II proof of concept results with Lu AG09222 in migraine prevention. Published 19 April 2023. Accessed 4 March 2024. https://news.cision.com/h--lundbeck-a-s/r/lundbeck-announces-positive-phase-ii-proof-of-concept-results-with-lu-ag09222-in-migraine-prevention,c3754245

[14] Headache Classification Committee of the International Headache Society (IHS) . The International Classification of Headache Disorders, 3rd edition. Cephalalgia. 2018; 38(1):1–211. 10.1177/0333102417738202 29368949

[15] Amin FM , Hougaard A, Schytz HW, et al Investigation of the pathophysiological mechanisms of migraine attacks induced by pituitary adenylate cyclase-activating polypeptide-38. Brain. 2014;137:779–794. 24501094 10.1093/brain/awt369

[16] Vollesen ALH , Snoer A, Chaudhry B, et al The effect of pituitary adenylate cyclase-activating peptide-38 and vasoactive intestinal peptide in cluster headache. Cephalalgia. 2020;40:1474–1488. 32962406 10.1177/0333102420940689

[17] Amin FM , Asghar MS, Guo S, et al Headache and prolonged dilatation of the middle meningeal artery by PACAP38 in healthy volunteers. Cephalalgia. 2012;32:140–149. 22174350 10.1177/0333102411431333

[18] Warren JB , Donnelly LE, Cullen S, et al Pituitary adenylate cyclase-activating polypeptide: A novel, long-lasting, endothelium-independent vasorelaxant. Eur J Pharmacol. 1991;197(2–3):131–134. 1915565 10.1016/0014-2999(91)90511-n

[19] Syed AU , Koide M, Braas KM, May V, Wellman GC. Pituitary adenylate cyclase-activating polypeptide (PACAP) potently dilates middle meningeal arteries: Implications for migraine. J Mol Neurosci. 2012;48:574–583. 22766684 10.1007/s12031-012-9851-0 PMC3581331

[20] Vaudry D , Falluel-Morel A, Bourgault S, et al Pituitary adenylate cyclase-activating polypeptide and its receptors: 20 years after the discovery. Pharmacol Rev. 2009;61:283–357. 19805477 10.1124/pr.109.001370

[21] Koide M , Syed AU, Braas KM, May V, Wellman GC. Pituitary adenylate cyclase activating polypeptide (PACAP) dilates cerebellar arteries through activation of large-conductance Ca2+-activated (BK) and ATP-sensitive (KATP) K+ channels. J Mol Neurosci. 2014;54:443–450. 24744252 10.1007/s12031-014-0301-z PMC4201911

[22] Nelson MT , Quayle JM. Physiological roles and properties of potassium channels in arterial smooth muscle. Am J Physiol. 1995;268(4 Pt 1):C799–C822. 7733230 10.1152/ajpcell.1995.268.4.C799

[23] Al-Karagholi MA , Hansen JM, Guo S, Olesen J, Ashina M. Opening of ATP-sensitive potassium channels causes migraine attacks: A new target for the treatment of migraine. Brain. 2019;142:2644–2654. 31292608 10.1093/brain/awz199

[24] Al-Karagholi MA , Ghanizada H, Hansen JM, et al Levcromakalim, an adenosine triphosphate-sensitive potassium channel opener, dilates extracerebral but not cerebral arteries. Headache. 2019;59:1468–1480. 31535367 10.1111/head.13634

[25] Al-Karagholi MA , Ghanizada H, Waldorff Nielsen CA, et al Opening of BKCa channels causes migraine attacks: A new downstream target for the treatment of migraine. Pain. 2021;162:2512–2520. 34252916 10.1097/j.pain.0000000000002238

[26] Al-Karagholi MA , Ghanizada H, Nielsen CAW, et al Opening of BK_Ca_ channels alters cerebral hemodynamic and causes headache in healthy volunteers. Cephalalgia. 2020;40:1145–1154. 32847403 10.1177/0333102420940681

[27] Ashina M . Migraine. N Eng J Med. 2020;383:1866–1876. 10.1056/NEJMra1915327 33211930

[28] Jansen-Olesen I , Hougaard Pedersen S. PACAP And its receptors in cranial arteries and mast cells. J Headache Pain. 2018;19:16. 29460121 10.1186/s10194-017-0822-2 PMC5818390

[29] Levy D , Edut S, Baraz-Goldstein R, et al Responses of dural mast cells in concussive and blast models of mild traumatic brain injury in mice: Potential implications for post-traumatic headache. Cephalalgia. 2016;36:915–923. 26566937 10.1177/0333102415617412 PMC5500910

[30] Levy D , Burstein R, Kainz V, Jakubowski M, Strassman AM. Mast cell degranulation activates a pain pathway underlying migraine headache. Pain. 2007;130:166–176. 17459586 10.1016/j.pain.2007.03.012 PMC2045157

[31] Pedersen SH , la Cour SH, Calloe K, et al PACAP-38 and PACAP(6–38) degranulate rat meningeal mast cells via the orphan MrgB3-receptor. Front Cell Neurosci. 2019;13:114. 30983973 10.3389/fncel.2019.00114 PMC6447718

[32] Krabbe AA , Olesen J. Headache provocation by continuous intravenous infusion of histamine. Clinical results and receptor mechanisms. Pain. 1980;8:253–259. 7402688 10.1016/0304-3959(88)90012-7

[33] Akerman S , Williamson DJ, Kaube H, Goadsby PJ. The role of histamine in dural vessel dilation. Brain Res. 2002;956:96–102. 12426051 10.1016/s0006-8993(02)03485-6

[34] Vollesen LH , Guo S, Andersen MR, Ashina M. Effect of the H_1_ -antihistamine clemastine on PACAP38 induced migraine. Cephalalgia. 2019;39:597–607. 30165750 10.1177/0333102418798611

[35] Bhatt DK , Gupta S, Olesen J, Jansen-Olesen I. PACAP-38 infusion causes sustained vasodilation of the middle meningeal artery in the rat: Possible involvement of mast cells. Cephalalgia. 2014;34:877–886. 24563332 10.1177/0333102414523846

[36] Zhang XC , Levy D. Modulation of meningeal nociceptors mechanosensitivity by peripheral proteinase-activated receptor-2: The role of mast cells. Cephalalgia. 2008;28:276–284. 18254896 10.1111/j.1468-2982.2007.01523.x PMC2504502

[37] Mason BN , Hassler SN, DeFea K, et al PAR2 Activation in the dura causes acute behavioral responses and priming to glyceryl trinitrate in a mouse migraine model. J Headache Pain. 2023;24:42. 37072694 10.1186/s10194-023-01574-5 PMC10114383

[38] Fujii N , McNeely BD, Zhang SY, Abdellaoui YC, Danquah MO, Kenny GP. Activation of protease-activated receptor 2 mediates cutaneous vasodilatation but not sweating: Roles of nitric oxide synthase and cyclo-oxygenase. Exp Physiol. 2017;102:265–272. 27981668 10.1113/EP086092

[39] Kopruszinski CM , Thornton P, Arnold J, et al Characterization and preclinical evaluation of a protease activated receptor 2 (PAR2) monoclonal antibody as a preventive therapy for migraine. Cephalalgia. 2020;40:1535–1550. 33131305 10.1177/0333102420966581

[40] Akerman S , Goadsby PJ. Neuronal PAC_1_ receptors mediate delayed activation and sensitization of trigeminocervical neurons: Relevance to migraine. Sci Transl Med. 2015;7:308ra157. 10.1126/scitranslmed.aaa7557 26446954

[41] Banks WA , Kastin AJ, Komaki G, Arimura A. Passage of pituitary adenylate cyclase activating polypeptide1–27 and pituitary adenylate cyclase activating polypeptide1-38 across the blood-brain barrier. J Pharmacol Exp Ther. 1993;267:690–696. 8246142

[42] Ashina M , Doležil D, Bonner JH, et al A phase 2, randomized, double-blind, placebo-controlled trial of AMG 301, a pituitary adenylate cyclase-activating polypeptide PAC1 receptor monoclonal antibody for migraine prevention. Cephalalgia. 2021;41:33–44. 33231489 10.1177/0333102420970889 PMC7786389

[43] Ernstsen C , Christensen SL, Rasmussen RH, et al The PACAP pathway is independent of CGRP in mouse models of migraine: Possible new drug target? Brain. 2022;145:2450–2460. 35136961 10.1093/brain/awac040

[44] Guo S , Jansen-Olesen I, Olesen J, Christensen SL. Role of PACAP in migraine: An alternative to CGRP? Neurobiol Dis. 2023;176:105946. 36481434 10.1016/j.nbd.2022.105946

[45] Ashina H , Iljazi A, Al-Khazali HM, et al Efficacy, tolerability, and safety of erenumab for the preventive treatment of persistent post-traumatic headache attributed to mild traumatic brain injury: An open-label study. J Headache Pain. 2020;21:62. 32493206 10.1186/s10194-020-01136-z PMC7271543

[46] Spierings ELH , Silberstein S, Najib U, et al A phase 2 study of fremanezumab as a treatment for posttraumatic headache in adult patients (1588). Neurology. 2021;96(15 Suppl).

[47] Navratilova E , Rau J, Oyarzo J, et al CGRP-dependent and independent mechanisms of acute and persistent post-traumatic headache following mild traumatic brain injury in mice. Cephalalgia. 2019;39:1762–1775. 31550910 10.1177/0333102419877662

