# Supplementary material for: Hypersensitivity to PACAP-38 in post-traumatic headache: a randomized clinical trial
Source: Brain. 2023 Oct 21;147(4):1312–20. doi: 10.1093/brain/awad367 (PMC10994530; doi:10.1093/brain/awad367)
Supplement: awad367_Supplementary_Data [file awad367_supplementary_data.zip › brain-2023-01311-File008_v2.pdf]

Supplementary Figure 1. Characteristics of Headache and Associated Symptoms at the Time of Peak Headache Intensity on PACAP-38 and Placebo Day.

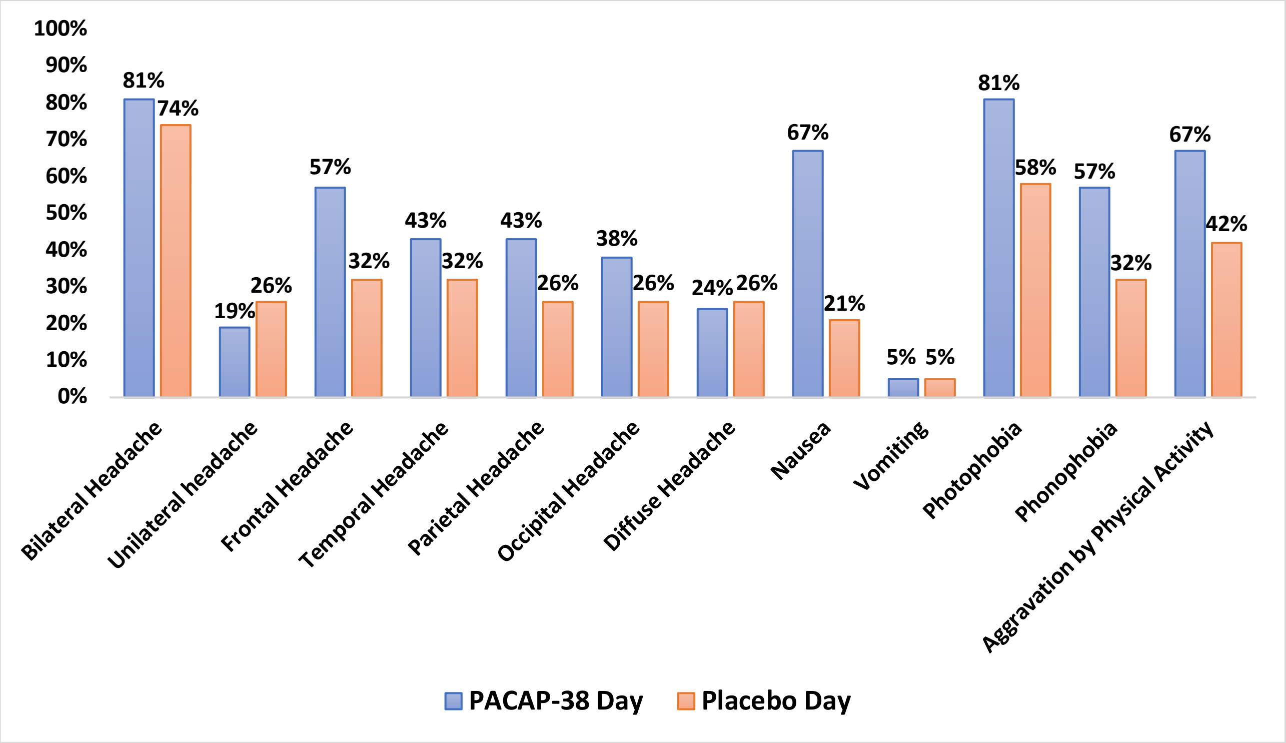

PACAP-38, pituitary adenylate cyclase-activating polypeptide-38.

Supplementary Table 1. Inclusion Criteria

Inclusion Criteria	Data Source
**Age 18 to 65 years of age upon entry into screening**	Legal identification document
**History of persistent headache attributed to mild traumatic injury to the head for ≥ 12 months and in accordance with the International Classification of Headache Disorders, 3rd Edition (ICHD-3)**	Medical record and/or subject self-report as assessed by site investigator during the semi-structured interview
**≥ 4 monthly headache days on average across the 3 months prior to screening**	Subject self-report as assessed by site investigator during the semi-structured interview
**Provision of informed consent prior to initiation of any study-specific activities/procedures.**	Informed consent form

Supplementary Table 2. Exclusion Criteria.

Exclusion Criteria	Data Source
**> 1 mild traumatic injury to the head**	Medical record and/or subject self-report as assessed by site investigator
**History of any primary or secondary headache disorder prior to mild traumatic injury to the head (except for infrequent episodic tension-type headache)**	Medical record and/or subject self-report as assessed by site investigator during the semi-structured interview
**History of moderate or severe injury to the head**	Medical record and/or subject self-report as assessed by site investigator during the semi-structured interview
**History of whiplash injury**	Medical record and/or subject self-report as assessed by site investigator during the semi-structured interview
**History of craniotomy**	Medical record and/or subject self-report as assessed by site investigator during the semi-structured interview
**History or evidence of any other clinically significant disorder, condition or disease (except for those outlined above) than, in the opinion of the site investigator, would pose a risk to subject safety or interfere with study evaluation, procedures or completion**	Medical record and/or subject self-report as assessed by site investigator during the semi-structured interview
**The subject is at risk of self-harm or harm to others as evidenced by past suicidal behavior**	Medical record and/or subject self-report as assessed by site investigator
**Female subjects of childbearing potential with a positive pregnancy test during any study visit**	Human chorionic gonadotropin (hCG) test (urine)
**Cardiovascular disease of any kind, including cerebrovascular diseases**	Medical record and/or subject self-report as assessed by site investigator during the semi-structured interview
**Hypertension (systolic blood pressure of ≥150 mmHg and/or diastolic blood pressure of ≥100 mmHg) prior to the start of infusion on the experimental day**	Blood pressure measurement

**Hypotension (systolic blood pressure of ≤90 mmHg and/or diastolic blood pressure of ≤50 mmHg)**	Blood pressure measurement
**Initiation, discontinuation, or change of dosing of prophylactic medications within 2 months prior to study inclusion**	Medical record and/or subject self-report as assessed by site investigator during the semi-structured interview
**Intake of acute medications (e.g. analgesics, triptans) within 48 hours of infusion start**	Subject self-report as assessed by site investigator during the semi-structured interview
**Baseline headache intensity of >3 on an 11-point numeric rating scale (0 being no headache, 10 being the worst imaginable headache)**	Subject self-report as assessed by site investigator during the semi-structured interview
**Baseline headache with migraine-like features or self-reported baseline headache that mimics the subjects' usual headache with migraine-like features**	Subject self-report as assessed by site investigator during the semi-structured interview

Supplementary Table 3 Characteristics of migraine-like headache after PACAP-38 and placebo

Participant no./sex	Headache phenotype	Type of intervention	Time to peak headache	Peak headache characteristic[a]	Mimics usual migraine-like headache[b]	Migraine-like headache[c] (time to onset)	Worsening of associated symptom[d]
1/Male	TTH-like	PACAP-38<br>Placebo<br>Usual	10 min<br>360 min	Bilat/7/Throb/−<br>Unilat/4/Pres/−<br>Bilat/5/Pres/−	No<br>No	Yes (120 min)<br>No	−/+/+<br>NA
2/Female	Migraine-like	PACAP-38<br>Placebo<br>Usual	10 min<br>0 min	Bilat/5/Throb/+<br>Bilat/3/Pres/+<br>Bilat/5/Comb/−	Yes<br>Yes	Yes (10 min)<br>No	+/+/+<br>NA
3/Female	Migraine-like	PACAP-38<br>Placebo<br>Usual	660 min<br>660 min	Bilat/7/Throb/+<br>Unilat/7/Pres/+<br>Bilat/9/Pres/+	Yes<br>No	Yes (10 min)<br>No	+/+/+<br>NA
4/Female	Migraine-like	PACAP-38<br>Placebo<br>Usual	20 min<br>NA	Bilat/6/Throb/−<br>NA<br>Unilat/7/Throb/+	Yes<br>NA	Yes (10 min)<br>NA	−/+/−<br>NA
5/Female	Migraine-like	PACAP-38<br>Placebo<br>Usual	40 min<br>480 min	Bilat/4/Pres/−<br>Bilat/4/Pres/−<br>Unilat/5/Pres/−	No<br>No	Yes (480 min)<br>No	+/−/−<br>NA
6/Male	Migraine-like	PACAP-38<br>Placebo<br>Usual	180 min<br>420 min	Bilat/7/Pres/+<br>Bilat/3/Pres/+<br>Bilat/7/Comb/+	Yes<br>No	Yes (120 min)<br>No	+/+/+<br>NA
7/Female	Migraine-like	PACAP-38<br>Placebo<br>Usual	300 min<br>420 min	Bilat/7/Pres/+<br>Bilat/3/Pres/+<br>Bilat/9/Throb/+	Yes<br>No	Yes (120 min)<br>No	+/+/+<br>NA
8/Female	Migraine-like	PACAP-38<br>Placebo<br>Usual	0 min<br>0 min	Bilat/3/Pres/−<br>Bilat/3/Pres/−<br>Bilat/4/Pres/+	Yes<br>Yes	Yes (10 min)<br>No	−/+/+<br>NA
9/Female	Migraine-like	PACAP-38<br>Placebo<br>Usual	240 min<br>40 min	Bilat/5/Pres/+<br>Bilat/2/Pres/−<br>Unilat/5/Pres/+	Yes<br>No	Yes (30 min)<br>No	+/+/+<br>NA
10/Female	Migraine-like	PACAP-38<br>Placebo<br>Usual	300 min<br>20 min	Unilat/9/Pres/+<br>Bilat/4/Pres/−<br>Bilat/6/Throb/+	Yes<br>No	Yes (10 min)<br>No	+/+/+<br>NA
11/Male	Migraine-like	PACAP-38<br>Placebo<br>Usual	660 min<br>NA	Bilat/5/Throb/−<br>NA<br>Bilat/3/Pres/−	No<br>NA	Yes (660 min)<br>NA	+/−/−<br>NA
12/Female	Migraine-like	PACAP-38<br>Placebo<br>Usual	360 min<br>60 min	Bilat/4/Pres/+<br>Unilat/1/Pres/−<br>Bilat/4/Comb/+	Yes<br>No	Yes (10 min)<br>No	+/+/+<br>NA
13/Female	Migraine-like	PACAP-38<br>Placebo<br>Usual	180 min<br>30 min	Unilat/5/Throb/+<br>Unilat/2/Pres/−<br>Unilat/7/Throb/+	Yes<br>No	Yes (20 min)<br>No	+/−/+<br>NA
14/Male	Migraine-like	PACAP-38<br>Placebo<br>Usual	30 min<br>540 min	Bilat/7/Throb/+<br>Unilat/7/Pres/+<br>Bilat/6/Throb/+	Yes<br>Yes	Yes (10 min)<br>Yes (480 min)	+/+/+<br>+/+/−
15/Female	Migraine-like	PACAP-38<br>Placebo<br>Usual	120 min<br>120 min	Bilat/6/Throb/+<br>Unilat/1/Throb/−<br>Bilat/6/Throb/+	Yes<br>No	Yes (20 min)<br>No	+/+/−<br>NA
16/Female	Migraine-like	PACAP-38<br>Placebo<br>Usual	180 min<br>0 min	Bilat/8/Pres/+<br>Unilat/1/Pres/−<br>Bilat/10/Throb/+	Yes<br>No	Yes (180)<br>No	+/+/+<br>NA

17/Female	Migraine-like	PACAP-38<br>Placebo<br>Usual	300 min<br>540 min	Bilat/3/Pres/−<br>Bilat/4/Pres/−<br>Bilat/7/Pres/+	No<br>No	No<br>No	NA<br>NA
18/Male	Migraine-like	PACAP-38<br>Placebo<br>Usual	240 min<br>300 min	Bilat/5/Pres/−<br>Bilat/7/Throb/+<br>Bilat/6/Throb/+	Yes<br>Yes	Yes (10 min)<br>Yes (30)	−/+/+<br>+/+/+
19/Female	TTH-Like	PACAP-38<br>Placebo<br>Usual	480 min<br>0 min	Unilat/6/Pres/+<br>Unilat/3/Pres/−<br>Bilat/4/Comb/+	No<br>No	Yes (180 min)<br>No	+/+/+<br>NA
20/Female	Migraine-like	PACAP-38<br>Placebo<br>Usual	420 min<br>0 min	Unilat/7/Throb/+<br>Bilat/3/Pres/−<br>Unilat/7/Throb/+	Yes<br>No	Yes (120 min)<br>No	+/+/+<br>NA
21/Female	Migraine-Like	PACAP-38<br>Placebo<br>Usual	120 min<br>180 min	Bilat/7/Throb/+<br>Unilat/6/Pres/+<br>Bilat/8/Comb/+	Yes<br>No	Yes (10 min)<br>No	+/+/+<br>NA

n = number; TTH = tension-type headache; PACAP-38 = pituitary adenylate cyclase-activating polypeptide-38; Bilat = bilateral; Throb = throbbing; Unilat = unilateral; Pres = pressing; NA = not applicable; Comb = combined throbbing and pressing quality of headache; + = present; − = absent.

[a]Localization (unilateral, bilateral) / pain intensity (11-point numeric rating scale, with 0 indicating no headache and 10 indicating the worst headache imaginable) / quality of headache (throbbing, pressing, combined throbbing and pressing) / aggravation of headache by routine physical activity (+ denotes presence, − denotes absence).

[b]The participant is asked to determine whether the headache following PACAP-38 or placebo infusion resembles his/her, if applicable, usual migraine-like headache.

[c]Migraine-like headache is defined by the criteria outlined in Box 1..

[d]Associated symptoms are nausea, photophobia, and phonophobia, respectively. Worsening is defined as an increase in the severity of these symptoms at the onset of migraine-like headache compared with baseline (i.e. time of infusion start), as rated on a 4-point Likert scale (0 = none, 1 = mild, 2 = moderate, 3 = severe).

Supplementary Table 4. Headache Intensity and Associated Symptoms at the Time of Infusion Start on PACAP-38 and Placebo Day.

Participant No./Sex	Baseline Headache Intensity[a] on PACAP-38 Day	Associated Symptoms[b] on PACAP-38 Day	Baseline Headache Intensity on Placebo Day	Associated Symptoms on Placebo Day
**1/Male**	2	Photophobia	3	Photophobia
**2/Female**	3	Photophobia, phonophobia	3	Photophobia, phonophobia
**3/Female**	2	Photophobia	2	Photophobia
**4/Female**	3	Photophobia	0	None
**5/Female**	2	None	2	None
**6/Male**	1	None	0	Photophobia
**7/Female**	2	None	2	Photophobia
**8/Female**	3	Photophobia, phonophobia	3	Photophobia, phonophobia
**9/Female**	1	Photophobia	1	None
**10/Female**	2	Nausea, photophobia	3	None
**11/Male**	0	None	0	None
**12/Female**	2	Nausea	0	Photophobia
**13/Female**	0	Nausea, photophobia	0	Photophobia
**14/Male**	2	None	1	Photophobia
**15/Female**	0	None	0	None
**16/Female**	2	None	1	None
**17/Female**	2	None	2	None
**18/Male**	3	Photophobia, phonophobia	3	Photophobia, phonophobia
**19/Female**	0	None	0	Photophobia
**20/Female**	2	None	3	None
**21/Female**	3	Photophobia, phonophobia	3	Photophobia

No, number; PACAP-38, pituitary adenylate cyclase-activating polypeptide-38.

11-point numeric rating scale (0 being no headache, 10 being the worst imaginable headache).

[a] Headache intensity is measured using a an 11-point numeric rating scale, with 0 indicating no headache and 10 indicating the worst headache imaginable.

[b] Associated symptoms are nausea, photophobia, and phonophobia, respectively.

Supplementary Table 5. Current Use of Acute and Preventive Headache Medication of the Study Population.

Participant No./Sex	Acute Headache Medication	Preventive Headache Medication
**1/Male**	Acetaminophen, Ibuprofen, Combination Analgesic (Acetaminophen, Caffeine, Acetylsalicylic Acid)	Amitriptyline
**2/Female**	Acetaminophen, Ibuprofen	Candesartan
**3/Female**	Sumatriptan	None
**4/Female**	Ibuprofen	Candesartan
**5/Female**	Acetaminophen, Ibuprofen	Erenumab
**6/Male**	Acetaminophen, Ibuprofen	None
**7/Female**	Acetaminophen, Sumatriptan	Nortriptyline
**8/Female**	Acetaminophen, Combination Analgesic (Acetaminophen, Caffeine, Acetylsalicylic Acid)	OnabotulinumtoxinA, Magnesium
**9/Female**	Acetaminophen, Ibuprofen, Combination Analgesic (Acetaminophen, Caffeine, Acetylsalicylic Acid), Eletriptan	Candesartan
**10/Female**	None	None
**11/Male**	Acetaminophen, Ibuprofen	None
**12/Female**	Acetaminophen, Ibuprofen	None
**13/Female**	Acetaminophen, Ibuprofen, Combination Analgesic (Acetaminophen, Caffeine, Acetylsalicylic Acid)	Candesartan
**14/Male**	Acetaminophen, Ibuprofen, Naproxen, Morphine	None
**15/Female**	Acetaminophen, Ibuprofen, Combination Analgesic (Acetaminophen, Caffeine, Acetylsalicylic Acid)	None
**16/Female**	Acetaminophen, Combination Analgesic (Acetaminophen, Caffeine, Acetylsalicylic Acid)	None
**17/Female**	Naproxen	Pregabalin
**18/Male**	None	Magnesium
**19/Female**	Acetaminophen, Ibuprofen	None
**20/Female**	Acetaminophen, Ibuprofen, Combination Analgesic (Acetaminophen, Caffeine, Acetylsalicylic Acid)	None
**21/Female**	None	None

No., number.
