# Supplementary material for: Hypersensitivity to PACAP-38 in post-traumatic headache: a randomized clinical trial
Source: Brain. 2023 Oct 21;147(4):1312–20. doi: 10.1093/brain/awad367 (PMC10994530; doi:10.1093/brain/awad367)
Supplement: awad367_Supplementary_Data [file awad367_supplementary_data.zip › brain-2023-01311-File009.pdf]

Hypersensitivity to PACAP-38 in Post-Traumatic Headache: A Randomized Clinical Trial.

Hypersensitivitet overfor PACAP-38 hos Folk kendt med Post-Traumatisk Hovedpine: Et Randomiseret Klinisk Forsøg.

PROTOCOL

March 5, 2022

VERSION 3

Corresponding Department:

Danish Headache Center, Rigshospitalet Glostrup, Valdemar Hansens Vej 5, Entrance 1A, DK-2600 Glostrup, Denmark.

Corresponding Doctor:

Håkan Ashina, MD

Danish Headache Center, Department of Neurology

Rigshospitalet Glostrup

Faculty of Health and Medical Sciences, University of Copenhagen

Valdemar Hansen Vej 5, Entrance 1A, DK-2600 Glostrup, Denmark

Tel: +45 28 10 24 95

E-mail: haakan.ashina@regionh.dk

Investigators

Table 1. List of Investigators and Collaborators.

Full Name	Role	Institution
Håkan Ashina, MD	Principal Investigator	Danish Headache Center, Rigshospitalet, Copenhagen, Denmark
Faisal M. Amin, MD	Investigator	Danish Headache Center, Rigshospitalet, Copenhagen, Denmark
Haidar M. Al-Khazali	Investigator	Danish Headache Center, Rigshospitalet, Copenhagen, Denmark
Rune H. Christensen	Investigator	Danish Headache Center, Rigshospitalet, Copenhagen, Denmark

Objective

To investigate whether PACAP-38 induces headache with migraine-like features in people with persistent post-traumatic headache (PTH) attributed to mild traumatic brain injury (mTBI).

Background

PTH is a disabling secondary headache disorder, which is often attributed to mTBI and affects millions of individuals worldwide (Ashina et al., 2021). Population-based data have shown that the lifetime prevalence of this disorder is estimated to be 4.7% in men and 2.4% in women (Rasmussen et al., 1992). Progress in research has improved disease characterization and shed light on the natural course of the disorder (Ashina et al., 2021). The clinical presentation of PTH is characterized by recurrent episodes of headache that can vary considerably in terms of frequency, duration, and pain intensity (Ashina et al., 2020). Headache features are often reported to resemble those of migraine (Ashina et al., 2020). A 2020 clinic-based study reported that 91 (91%) of 100 patients with persistent PTH and no pre-existing primary headache disorder (except infrequent episodic tension-type headache) had recurrent headache episodes with migraine-like features (Ashina et al., 2020). This observation raises questions about whether PTH and migraine share similar mechanisms, perhaps within the framework of a final common pathway implicated in headache pathogenesis (Ashina et al., 2021).

The trigeminovascular system is posited to be the anatomical and physiological substrate of headache pathogenesis, incl. migraine and PTH (Ashina et al., 2019). Axonal projections arise from trigeminal ganglion cells to innervate pain-sensitive intracranial structures, e.g., meninges—dura mater and pial arteries (Ashina et al., 2019). These innervating nerve fibers contain vasoactive signaling molecules that, upon release, modulate nociceptive transmission and promote vasodilation. These signaling molecules include calcitonin gene-related peptide (CGRP) and pituitary adenylate cyclase-activating polypeptide-38 (PACAP-38). It has been demonstrated that intravenous infusion of CGRP induces migraine attacks in people with migraine (Hansen et al., 2010), whereas people with persistent PTH (and no pre-existing migraine) develop headache exacerbation with migraine-like features (Ashina et

al., 2021). Interestingly, CGRP mediates its effects on the vascular smooth muscle cells within the walls of intracranial arteries and result in opening of potassium channels and accompanying vasodilation (Amin et al., 2012). This, in turn, is hypothesized to provide the necessary mechanical and chemical stimuli to activate and sensitize the afferent nociceptive fibers that project to first order neurons in the trigeminal ganglion. The ascending nociceptive transmission is ultimately relayed to the somatosensory cortex and yields headache.

An outstanding scientific question, that merits investigation, is whether PACAP-38 is implicated in the pathogenesis of headache with migraine-like features in persistent PTH, just as in migraine (Amin et al., 2014). Here, it should also be noted that intravenous infusion of PACAP-38 induces transient mild headache in healthy volunteers but not migraine attacks (Schytz et al., 2009).

We hypothesize that intravenous infusion of PACAP-38 induces headache with migraine-like features in people with persistent PTH (and no pre-existing migraine). To test this hypothesis, we will conduct a randomized, double-blind, placebo-controlled, 2-way crossover study.

Outcomes

Primary Outcome:

- Difference in incidence of headache with migraine-like features* (0 to 12 hours) between PACAP-38 and placebo.

*The following definition of headache with migraine-like features will be used:

Migraine-like features are defined as headache fulfilling at least two of the following four characteristics:

- Unilateral location,
- Pulsating quality,
- Moderate to severe pain intensity, and
- Aggravation by causing avoidance of routine physical activity (e.g., walking or climbing stairs); and

During headache, at least one of the following must be fulfilled:

- Nausea and/or vomiting,
- Photophobia and phonophobia, and
- Headache mimicking the usual headache with migraine-like features

Secondary Outcome:

- Difference in area under the curve (AUC) for headache intensity scores (0 to 12 hours) between PACAP-38 and placebo.

Study Design

Study participants will be referred to as “subjects”.

Subjects will be randomly allocated to receive continuous intravenous infusion of 10 pmol/kg/min PACAP-38 (Bachem AG, Bubendorf, Switzerland) or 20 ml placebo (isotonic saline) over 20 min on 2 experimental days that will be separated by at least 1 week. The doses are identical to those that were used in previous experimental studies that included subjects with migraine without aura, as well as healthy volunteers free of migraine and frequent headache (Amin et al., 2012; Amin et al., 2014). A block randomization method is used to randomize subjects into groups that result in equal sample

sizes. Sealed envelopes will be provided with randomization codes that will not disclosed until study completion.

Study Flow

Study arrival is set to between 08:00 AM and 12:00 PM on both experimental days. Before the start of infusion, site investigators will perform a physical examination (incl. neurological examination). Baseline is defined as the time of infusion start. For both experimental days, study eligibility is contingent on no intake of acute medications within 48 hours of infusion start and a baseline headache intensity of ≤3 on an 11-point numeric rating scale (0 being no headache, 10 being the worst imaginable headache). If a subject reports a baseline headache, that mimics their usual headache with migraine-like features, the experimental day must be rescheduled. On experimental day 1, an in-person semi-structured interview will also be carried out record data on demographics, medical history, and full clinical course.

A time- and volume-controlled infusion pump is used to administer PACAP-38 or placebo (isotonic saline) over 20 minutes. At baseline on both experimental days, site investigators record data on headache characteristics, vital signs, and adverse events. This procedure is repeated every 10 minutes until 60 minutes after infusion start. Thereafter, patients are discharged with a headache diary that they have to complete every hour until 12 hours after the start of infusion.

Table 1. Study Overview.

**Study Type**	**Interventional (Clinical Trial)**
**Estimated Enrollment**	21
**Allocation**	Randomized

**Intervention Model**	Crossover Assignment
**Masking**	Quadruple
**Expected Study Start Date**	May 1, 2022
**Expected Study Completion Date**	January 1, 2027

Table 2. Overview of Study Arms and Interventions.

**Arm**	**Intervention**
Active Comparator: PACAP-38	Drug: PACAP-38<br>Administered by intravenous infusion
Placebo Comparator: Isotonic Saline	Drug: Isotonic Saline<br>Administered by intravenous infusion

Subject Recruitment and Enrollment

Recruitment

The principal investigator will contact physicians in charge of treating patients at the outpatient clinic of the Danish Headache Center. The physicians will be requested to ask potential subjects during their daily consultations in the clinic if they are allowed to pass on (Danish: 'videregive') the patients' contact information to the study investigators (Appendix 1). If the patient agrees, he/she will be contacted within four weeks by a site investigator to provide oral study information and the following written information about study participation: (1) subject participation folder (Appendix 2), (2) the pamphlet 'Før du beslutter dig – om at være forsøgsperson i sundhedsvidenskabelige forsøg', and (3) the pamphlet 'Forsøgspersoners rettigheder i et sundhedsvidenskabeligt forskningsprojekt'. Potential subjects will be encouraged to read all three documents and must have at least three days to consider whether they would like to participate in the study. Potential subjects will also be informed by phone

that they can bring, if preferred, an external assessor (Danish: 'bisidder') to be present during the initial site visit and all study-related activities or procedures. At the initial site visit, potential subjects will again receive oral information about the study in an uninterrupted environment and then be asked to personally sign and date the informed consent form (ICF). It merits emphasis that potential subjects must have at least 3 days of reflection time (Danish: 'betænkningstid') from the day that they received oral and written study information till the day on which they sign the ICF.

Enrollment

All subjects must personally sign and date the ICF before the commencement of study-specific activities or procedures (see Appendix 3). A subject is considered enrolled when the site investigator decides that the subject meets all eligibility criteria. The investigator is to document this decision in the subject's medical records and in the enrollment case report form (CRF), (Appendix 4).

All subjects, who are enrolled (i.e. subjects with a signed ICF), will receive a unique subject identification number before any study-specific activities or procedures are performed. This subject identification number will be used to identify the subject throughout the study and must be used on all study documentation related to that subject. A potential subject who is deemed ineligible must be registered as a screen failure in the screening log.

Screen Failures

Screen failures are defined as subjects who consent to participate in the clinical study but are not subsequently enrolled in the study. A minimal set of screen failure information will be collected that includes demographic data and reason for study exclusion.

Subjects

Number of Subjects

A total of 21 subjects will be enrolled in the study.

Replacement of Subjects

Subjects, who are withdrawn or removed from study participation before completion of both experimental days, will be replaced.

Number of Sites

The study will be conducted at one study site, being the Danish Headache Center, Rigshospitalet Glostrup.

End of Study

Primary Completion: The primary completion date is defined as the date when a subject has completed both experimental days or withdraws from study participation.

End of Study: The end of study is defined as the date when the last subject is assessed for the final collection of data for the primary outcome. If the study is terminated early, then the end of the study will be the date when the last subject is assessed or receives an intervention (i.e. last site visit).

Subject Eligibility

Site investigators must maintain a screening log of all potential subjects that includes limited information, such as the date of the screening (Appendix 5). Before any study-specific activities or procedures are performed, investigators must obtain written informed consent (Appendix 3).

Inclusion Criteria

Subjects are eligible for inclusion if all of the following criteria apply:

Table 3. Inclusion Criteria.

Inclusion Criteria	Data Source
**Age 18 to 65 years of age upon entry into screening**	Legal identification document
**History of persistent headache attributed to mild traumatic injury to the head for ≥ 12 months and in accordance with the International Classification of Headache Disorders, 3rd Edition (ICHD-3)**	Medical record and/or subject self-report as assessed by site investigator during the semi-structured interview
**≥ 4 monthly headache days on average across the 3 months prior to screening**	Subject self-report as assessed by site investigator during the semi-structured interview
**Provision of informed consent prior to initiation of any study-specific activities/procedures.**	Informed consent form

Exclusion Criteria

Subjects are excluded from the study if any of the following criteria apply:

Table 4. Exclusion Criteria.

Exclusion Criteria	Data Source
**> 1 mild traumatic injury to the head**	Medical record and/or subject self-report as assessed by site investigator

**History of any primary or secondary headache disorder prior to mild traumatic injury to the head (except for infrequent episodic tension-type headache)**	Medical record and/or subject self-report as assessed by site investigator during the semi-structured interview
**History of moderate or severe injury to the head**	Medical record and/or subject self-report as assessed by site investigator during the semi-structured interview
**History of whiplash injury**	Medical record and/or subject self-report as assessed by site investigator during the semi-structured interview
**History of craniotomy**	Medical record and/or subject self-report as assessed by site investigator during the semi-structured interview
**History or evidence of any other clinically significant disorder, condition or disease (except for those outlined above) than, in the opinion of the site investigator, would pose a risk to subject safety or interfere with study evaluation, procedures or completion**	Medical record and/or subject self-report as assessed by site investigator during the semi-structured interview
**The subject is at risk of self-harm or harm to others as evidenced by past suicidal behavior**	Medical record and/or subject self-report as assessed by site investigator
**Female subjects of childbearing potential with a positive pregnancy test during any study visit**	Human chorionic gonadotropin (hCG) test (urine)
**Cardiovascular disease of any kind, including cerebrovascular diseases**	Medical record and/or subject self-report as assessed by site investigator during the semi-structured interview

**Hypertension (systolic blood pressure of ≥150 mmHg and/or diastolic blood pressure of ≥100 mmHg) prior to the start of infusion on the experimental day**	Blood pressure measurement
**Hypotension (systolic blood pressure of ≤90 mmHg and/or diastolic blood pressure of ≤50 mmHg)**	Blood pressure measurement
**Initiation, discontinuation, or change of dosing of prophylactic medications within 2 months prior to study inclusion**	Medical record and/or subject self-report as assessed by site investigator during the semi-structured interview
**Intake of acute medications (e.g. analgesics, triptans) within 48 hours of infusion start**	Subject self-report as assessed by site investigator during the semi-structured interview
**Baseline headache intensity of >3 on an 11-point numeric rating scale (0 being no headache, 10 being the worst imaginable headache)**	Subject self-report as assessed by site investigator during the semi-structured interview
**Baseline headache with migraine-like features or self-reported baseline headache that mimics the subjects' usual headache with migraine-like features**	Subject self-report as assessed by site investigator during the semi-structured interview

Study Procedures and Assessments

Study procedures and assessments are defined as any intended examinations of a study subject according to the study protocol (Table 5).

Table 2. Study Procedures and Assessments.

	**Experimental Day 1**	**Experimental Day 2**
Informed Consent Form	X	
Medical and Medication History	X	X
Physical Examination	X	X
Physical Measurements	X	X
In-Person Semi-Structured Interview	X	
hCG Urine Test	X	X
Blood Pressure Monitor	X	X
Peripheral Intravenous Cannulation	X	X
12-Hour Headache Diary	X	X

Site investigators are responsible for ensuring that all study procedures are performed as detailed in the protocol.

Informed Consent

All subjects must personally sign and date the ICF before any study-specific activities or procedures are performed.

Medical and Medication History

A review of medical and medication history will be performed by the site investigator prior to the start of infusion on both experimental days to confirm subject eligibility. These data are to be recorded in the Medical and Medication History CRF (Appendix 6).

Physical Examination

A complete physical examination per standard of care (incl. neurological examination) will be performed by the site investigator on all subjects. Any clinically significant anomalies noted during

the initial screening phase are to be detailed in the Medical and Medication History CRF (Appendix 6).

Physical Measurements

The following measurements are to be performed:

- Height (first experimental day only)
- Body weight

Height and weight are to be measured without shoes.

All measurements are to be recorded in the Medical and Medication History CRF (Appendix 6).

In-Person Semi-Structured Interview

An in-person semi-structured interview will be used to record data on demographics, medical history, and full clinical course (Appendix 7).

hCG Urine Test

Female subjects of childbearing potential must have a hCG urine test performed to confirm study eligibility prior to the start of infusion on both experimental days. The results of the pregnancy test will be recorded in the Medical and Medication History CRF (Appendix 6) .

Blood Pressure Monitor

A blood pressure monitor will be used to record systolic pressure, diastolic pressure, and heart rate.

Peripheral Intravenous Cannulation

A plastic catheter (cannula) will be inserted into a peripheral vein to gain venous access for the intravenous infusion of PACAP-38 or isotonic saline.

12-Hour Headache Diary

A 12-hour headache diary will be used to record data on headache characteristics, vital signs, and adverse events (Appendix 8). This procedure will be repeated every 10 minutes until 60 minutes after the start of infusion. Thereafter, patients will be discharged with a headache diary that they have to complete every hour until 12 hours after the start of infusion.

Discontinuation Criteria

Subjects' Decision to Withdraw

Subjects have the right to withdraw from the study at any time and for any reason without prejudice to their future medical care by site investigators or the study site. Subjects can decline to continue in any protocol-required procedures at any time during the study. Withdrawal of consent means that the subject does not wish to continue with protocol-required procedures, and the subject does not wish to or is unable to continue further study participation. Subject data up to withdrawal of consent will be included in the analysis of the study, and where permitted, publicly available data can be included after withdrawal of consent. The investigator is to discuss with the subject appropriate procedures for withdrawal from the study.

Investigator Decision to Withdraw or Terminate Subjects' Participation Prior to Study Completion

The investigator can decide to withdraw a subject from specific study procedures or the study as a whole at any time prior to study completion.

Statistical Considerations

Sample size calculations are based on the one-sided McNemar's Test that can be used to evaluate difference between two paired groups reporting headache with migraine-like features. Based on previous human provocation studies, we assume that 50% of subjects with persistent PTH will report headache with migraine-like features exclusively after PACAP-38 and 10% exclusively after placebo. At 80% power and a 5% level of significance, we estimate that inclusion of 21 subjects is needed for a two-way crossover superiority trial

Risk Assessment

The study will provide considerable neurobiological insights into the pathogenesis of PTH and holds promise to identify blockers of PACAP signaling as a novel drug target for PTH. Potential study-related risks are considered very mild and transient, as detailed below.

PACAP-38

PACAP-38 is a vasoactive substance that has been used in various human experimental studies (Amin et al., 2012; Amin et al., 2014). PACAP-38 causes outflow of potassium from the vascular smooth muscle cells (Amin et al., 2012). Thus, the cell becomes less active (hyperpolarized) causing blood vessel expansion (Amin et al., 2012). In healthy volunteers, the known side effects are transient headache of mild intensity (100%), flushing (100%), palpitations (92%), heat sensations (92%), and

nausea (42%), (Schytz et al., 2009).If a subject reports headache after the start of infusion, he/she will be offered, if needed, 50-mg oral sumatriptan to treat their headache during the in-hospital phase or at home.

Peripheral Intravenous Cannulation

Peripheral intravenous cannulation can cause transient unpleasant sensation at the insertion site and in some instances leave a subcutaneous hematoma that disappear within days. The procedure does not present with any serious adverse effects on health and wellbeing.

Regulatory Obligations

Informed Consent

Before a subject's participation in the clinical study, the investigator is responsible for obtaining written informed consent from the subject after adequate explanation of the aims, methods, anticipated benefits, and potential hazards of the study and before any protocol-specific screening procedures or any investigational product is administered. The original signed informed consent form is to be retained in accordance with institutional policy, and a copy of the signed consent form is to be provided to the subject.

Independent Ethics Committee

A copy of the protocol, informed consent form, other written subject information, and any proposed advertising material must be submitted to the institutional ethics committee (IEC) for written approval. The principal investigator must submit and, where necessary, obtain approval from the IEC for all subsequent protocol amendments and changes to the informed consent document. The principal investigator is to notify the IEC of deviations from the protocol or serious adverse events occurring

at the site and other adverse event reported. The principal investigator is responsible for obtaining annual IEC approval throughout the duration of the study.

PACAP-38 has previously been used in several human experimental studies at the Danish Headache Center (Amin et al., 2012; Amin et al., 2014). It merits emphasis that PACAP-38 is exclusively used as an experimental tool for the purposes of inducing headache in order to study underlying disease mechanisms. PACAP-38 is not used for therapeutic purposes. Based on this information, experimental use of PACAP-38 is not subject to notification of the Danish Medicines Agency (see the Danish Medicines Agency's guidance on the notification of clinical trials of medicinal products on humans). People with persistent PTH frequently report a migraine-like headache which is why PACAP-38 was chosen as an experimental tool in order to explore the involvement of PACAP-38 in the pathogenesis of PTH.

Danish Data Protection Agency and Processing of Personal Data

Processing of personal data will be observed in accordance with the law and data protection rules. All data collected will be treated confidentially and only published in anonymized form. Raw data and randomization codes are kept in anonymous form under safe conditions for 15 years after the end of the study. Personal data will be dealt with in accordance with the Regulation (EU) 2016/679 of the European Parliament and of the Council of 27 April 2016. All data will be obtained in accordance with the Health Act (Sundhedslovens § 46, stk. 1) and to the Data Protection Act and Regulation (Databeskyttelsesloven og -forordningen). The information will be used to screen the patient for inclusion and exclusion criteria related to the study.

Subject Confidentiality

The investigator must ensure that the subject's confidentiality is maintained. All subjects are to be provided a unique subject identification number. All documents with subject information are to be kept in confidence by the investigator and no subject-level information will be shared with third parties.

Administrative and Legal Obligations

Study Documentation and Archive

The investigator is to maintain a list of appropriately qualified persons to whom he/she has delegated study duties. Source documents are original documents, data, and records from which the subject's CRF data are obtained. These include but are not limited to hospital records, clinical and office charts, laboratory and pharmacy records, diaries, microfiches, radiographs, and correspondence. CRF entries may be considered source data if the CRF is the site of the original recording (i.e., there is no other written or electronic record of data). The Investigator and study staff are responsible for maintaining a comprehensive and centralized filing system of all study-related (essential) documentation.

Elements to include:

- Subject files containing completed CRFs, informed consent forms, and subject identification list
- Study files containing the protocol with all amendments, Investigator's Brochure, copies of pre-study documentation, and all correspondence to and from the IRB

In addition, all original source documents supporting entries in the CRFs must be maintained and be readily available.

Study Monitoring and Data Collection

Data capture for this study is planned to be electronic:

- All source documentation supporting entries into the CRFs must be maintained and readily available.
- Data will be stored in a secure server maintained by the Capital Region of Denmark.

Publication Policy

Publication activities will be undertaken responsibly and ethically to ensure that all relevant information is communicated clearly and in a timely manner. Per current Good Publication Practice, we will submit for publication the results of the study, primarily in peer-reviewed journals, or as abstracts, posters, or other presentations at scientific meetings. Results will be published irrespective of whether they are positive, negative, or inconclusive. Publications will be prepared in accordance with the guidelines established by the International Committee of Medical Journal Editors (ICMJE). Thus, we are committed to ensuring that authorship for all publications complies with the criteria developed by the ICMJE.

Unintended Health Care Errors and Insurance

All unintended events during the study are recorded and if serious unintended events. In this context, an unintended event is defined as: Any undesired event that is temporarily related to a study procedure, whether or not this accidental event is considered to be associated with the study procedure. If an unintended event occurs more than 14 days after a procedure, and there is no apparent causal link or association with the procedure, this is not considered an unintended event. In this context, a serious accidental incident means any incident which: results in death, is life threatening,

involves hospitalization or extension of existing hospitalization, results in persistent or significant disability / incapacity, or is a congenital anomaly / malformation. The study falls within patient insurance coverage of Rigshospitalet Glostrup.

Subject Compensation

Subject participation reimbursement is set at 900 DKK and is contingent upon completion of both experimental days (taxable B-income). Transport expenses up to 1,000 DKK will also be reimbursed (taxable B-income). If a subject withdraws from study participation prior to completion of the second experimental day, he/she will be reimbursed for transport expenses up to 1,000 DKK and receive a subject participation reimbursement set at 200 DKK.

Funding and Budget

The project is commenced on initiative of the Danish Headache Center, Department of Neurology Rigshospitalet Glostrup by Dr. Håkan Ashina, MD. The project is funded with 200.000 DKK provided by an unrestricted research grant from the Lundbeck Foundation. The investigators will apply for future funding as the study proceeds. If any additional funding is obtained, the study participants and the relevant ethics committee will be informed. The funding is in fully transferred to an account directed by the Capital Region which is subject to public audit. The funding sources will appear in the subject participation folder. The site investigators have no financial affiliation to the Lundbeck Foundation or other potential stakeholders.

References

Ashina H, Eigenbrodt AK, Seifert T, Sinclair AJ, Scher AI, Schytz HW, Lee MJ, De Icco R, Finkel AG, Ashina M. Post-traumatic headache attributed to traumatic brain injury: classification, clinical characteristics, and treatment. Lancet Neurol. 2021 Jun;20(6):460-469. doi: 10.1016/S1474-4422(21)00094-6. PMID: 34022171.

Rasmussen BK, Olesen J. Symptomatic and nonsymptomatic headaches in a general population. Neurology. 1992 Jun;42(6):1225-31. doi: 10.1212/wnl.42.6.1225. PMID: 1603351.

Ashina H, Iljazi A, Al-Khazali HM, Ashina S, Jensen RH, Amin FM, Ashina M, Schytz HW. Persistent post-traumatic headache attributed to mild traumatic brain injury: Deep phenotyping and treatment patterns. Cephalalgia. 2020 May;40(6):554-564. doi: 10.1177/0333102420909865. Epub 2020 Feb 26. PMID: 32102546.

Ashina H, Porreca F, Anderson T, Amin FM, Ashina M, Schytz HW, Dodick DW. Post-traumatic headache: epidemiology and pathophysiological insights. Nat Rev Neurol. 2019 Oct;15(10):607-617. doi: 10.1038/s41582-019-0243-8. Epub 2019 Sep 16. PMID: 31527806.

Amin FM, Asghar MS, Guo S, Hougaard A, Hansen AE, Schytz HW, van der Geest RJ, de Koning PJ, Larsson HB, Olesen J, Ashina M. Headache and prolonged dilatation of the middle meningeal artery by PACAP38 in healthy volunteers. Cephalalgia. 2012 Jan;32(2):140-9. doi: 10.1177/0333102411431333. Epub 2011 Dec 15. PMID: 22174350.

Amin FM, Hougaard A, Schytz HW, Asghar MS, Lundholm E, Parvaiz AI, de Koning PJ, Andersen MR, Larsson HB, Fahrenkrug J, Olesen J, Ashina M. Investigation of the pathophysiological

mechanisms of migraine attacks induced by pituitary adenylate cyclase-activating polypeptide-38. Brain. 2014 Mar;137(Pt 3):779-94. doi: 10.1093/brain/awt369. Epub 2014 Feb 5. PMID: 24501094.

Appendix

Appendix 1: 'Kontaktoplysninger'

Please see the attached file 'Kontaktoplysninger'.

Appendix 2: Subject Participation Folder

Please see the attached file 'Subject Participation Folder'.

Appendix 3: Informed Consent Form

Please see the attached file 'Informed Consent Form'.

Appendix 4: Enrollment Case Report Form

Please see the attached file 'Enrollment Case Report Form'.

Appendix 5: Screening Log

Please see the attached file 'Screening Log'.

Appendix 6: Medical and Medication Case Report Form

Please see the attached file 'Medical and Medication Case Report Form'.

Appendix 7: Semi-Structured Interview

Please see the attached file 'Semi-Structured Interview'.

Appendix 8: 12-Hour Headache Diary

Please see the attached file ’12-Hour Headache Diary’.

Appendix 9: Protokolresumé

Please see the attached file ‘Protokolresumé’.

Appendix 10: Sundhedskort

Please see the attached file ‘Sundhedskort’.

Appendix 11: Autorisations_Id

Please see the attached file ‘Autorisations_Id’.

Appendix 12: Curriculum Vitae

Please see the attached file ‘Curriculum Vitae’.
